# Satisfaction with cancer screening programs and its associated factors: a cross-sectional study in 26 provinces across China

**DOI:** 10.1186/s12889-026-27706-5

**Published:** 2026-05-21

**Authors:** Yuqian Zhang, Baokun Cao, Pengyun Meng, Zhicheng Wang, Haomin Yang

**Affiliations:** 1 Department of Epidemiology and Health Statistics, School of Public Health, Fujian Medical University, Xue Yuan Road 1, University Town, Fuzhou, 350122 China; 2 China Development Research Foundation, Beijing, China; 3 Department of Medical Epidemiology and Biostatistics, Karolinska Institutet, Stockholm, Sweden

**Keywords:** Cancer screening, Satisfaction, Associated factors

## Abstract

**Background:**

Satisfaction is a crucial indicator for evaluating cancer screening programs. However, the current levels of satisfaction with cancer screening services in China and the associated factors remain unclear.

**Methods:**

A national survey was conducted in 2022, including 5,024 participants aged 35 to 69 years who were involved in cancer screening programs across 26 provinces in China. Logistic regression models were used to investigate the associations between socioeconomic factors, lifestyle factors, and individual health status with satisfaction regarding cancer screening. Multilevel logistic regression was performed to examine provincial-level contextual factors and quantify regional disparities. Additionally, structural equation models were utilized to examine the pathways associated with screening satisfaction.

**Results:**

Overall, 82.8% of participants were satisfied with cancer screening. Provinces with relatively high satisfaction rates included Gansu (91%), Shandong (92%), and Anhui (87%), while provinces with relatively low satisfaction rates included Hainan (67%), Jiangxi (76%), and Jilin (75%). Independent factors associated with satisfaction were physical activity (OR = 1.27, 95% CI = 1.08–1.48), and self-rated health (OR for excellent health status = 4.51, 95% CI: 2.35–8.65; trend *P* < 0.001). For free screenings, independent factors were physical activity (OR = 1.45, 95% CI: 1.14–1.84) and family history of cancer (OR = 0.63, 95% CI: 0.48–0.84), with self-rated health showing a positive trend (trend *P* < 0.001). For paid screenings, independent factor was self-rated health (OR for excellent health status = 3.20, 95% CI: 1.62–6.34; trend *P* < 0.001). Multilevel modeling revealed that provincial-level differences accounted for only 1.4% of the total variance in satisfaction (ICC = 1.4%). None of the measured regional contextual factors (GDP per capita, hospital bed density, or number of tertiary hospitals) were significantly associated with satisfaction (all *P* > 0.05). Furthermore, self-rated health partially mediated the association between household income and satisfaction. In paid screening programs, chronic disease and service expectations also served as mediators.

**Conclusions:**

Satisfaction with cancer screening is generally high in China, although regional disparities persist. Provincial-level differences accounted for only a small proportion of the total variance, with individual-level factors being the primary drivers of satisfaction. Household income indirectly influences cancer screening satisfaction through its association with reduced chronic disease burden and improved self-rated health. Additional attention should be directed toward participants with unhealthy lifestyles and those with lower household income to effectively enhance overall satisfaction.

**Supplementary Information:**

The online version contains supplementary material available at 10.1186/s12889-026-27706-5.

## Introduction

In recent years, both the incidence and mortality rates of cancer have steadily increased worldwide [1]. In China, approximately 4.82 million new cancer cases and 2.57 million deaths were reported in 2022, and the disease burden continues to rise [2]. Cancer poses a significant threat to public health and has become one of the major challenges affecting individuals’ quality of life [1, 3].

Early screening and detection are essential strategies for reducing cancer incidence and mortality, improving the five-year survival rates of cancer patients, and significantly alleviating the disease burden associated with cancer [4]. Cancer screening has long been recognized as a critical component of cancer prevention. Moreover, satisfaction with cancer screening is important because it affects screening participation rates, clinical diagnoses, patient outcomes, and adherence to counseling and follow-up recommendations [5, 6]. Lower satisfaction has been associated with reduced return rates for screening and poor compliance with follow-up guidelines [7]. However, satisfaction with cancer screening and its associated factors have not yet been evaluated at the national level in China.

Some studies have indicated that satisfaction with cancer screening may be influenced by factors such as gender, place of residence, education, occupation, marital status, body mass index (BMI), type of screening organization, self-rated health status, and family history of cancer and other chronic diseases [8–10]. However, these studies are limited by small sample sizes and insufficient population representation. Furthermore, the mechanisms through which these factors affect satisfaction have not been thoroughly investigated.

To date, no nationally representative study has quantified satisfaction with cancer screening or examined its determinants in China, limiting evidence available for policy improvement. This study aims to identify the factors associated with participants’ satisfaction with cancer screening at the national level in China. It also investigates the underlying mechanisms linking these factors to satisfaction, and explores the associations between socioeconomic status, health behaviors, health status, and satisfaction with cancer screening.

## Materials and methods

### Study population

This study was conducted as part of the 2022 Chinese Residents Cancer Screening Services Survey, which included a total of 10,000 urban and rural residents aged 35 to 69 years across 26 provinces in China, utilizing a three-stage cluster sampling design: (1) purposive selection of 26 provinces and stratified them according to the economic development levels and geographic regions of China; (2) probability proportional to size (PPS) sampling of 100 urban districts and rural counties within selected provinces and 1000 urban communities or rural villages within selected districts/counties; and (3) recruitment of eligible residents aged 35–69 years using simple random sampling. The stratified clusters included: first-tier cities (Beijing, Shanghai, Guangzhou and Shenzhen), eastern regions, middle regions, western regions and northeastern regions. Five western regions (Inner Mongolia, Tibet, Xinjiang, Qinghai, and Ningxia) were excluded due to low population density and limited accessibility; Hong Kong, Macau, and Taiwan were also excluded because of their distinct healthcare systems. All participants were surveyed through face-to-face interviews conducted by trained interviewers. The survey collected data on basic personal characteristics (gender, BMI, education level, employment status, marital status, family history of cancer, and annual disposable income), lifestyle factors (smoking, alcohol consumption, vegetable intake, fruit intake, and physical activity), personal health status (history of chronic diseases and self-rated health status), and experiences with cancer screening (cost, organizers, and satisfaction with the cancer screening program). Of the 10,000 participants, individuals were excluded if they: (1) had never undergone cancer screening; (2) provided incomplete responses to key questionnaire items, including body mass index (BMI), annual household disposable income, and satisfaction with cancer screening; or (3) gave logically inconsistent answers. After applying these exclusion criteria, a final sample of 5,024 participants was included in the analysis.

This study was approved by the Ethics Committee of the National Cancer Center/Cancer Hospital of Peking Union Medical College of the Chinese Academy of Medical Sciences (ethical approval number: 22/360–3562), and all the participants provided written informed consent.

### Definition of variables

In this study, BMI was calculated as weight (kg) / height (m²) [11]. According to Chinese criteria, a BMI ranging from 18.5 kg/m² to less than 24.0 kg/m² is considered normal. A BMI below 18.5 kg/m² is classified as underweight, while a BMI of 24.0 kg/m² or greater is classified as overweight or obese. Smoking was defined as current smoking of at least 1 cigarette per day for a minimum of 6 months, adapted from the WHO definition of a smoker [12]. Passive smoking refers to non-smokers inhaling smoke from burning cigarettes or exhaled smoke from smokers on at least one day per week, for more than 15 min per day. Alcohol consumption is defined as drinking at least once per week for more than six months, regardless of the type of alcohol consumed (occasional drinking during New Year’s or holidays does not qualify). Physical activity was defined as engaging in physical activity at least three times per week on average, with each session lasting more than 30 min [13]. A family history of cancer is defined as the presence of malignant cancer in first-degree relatives, including biological parents, biological siblings, and children. Both personal annual disposable income and annual household disposable income were classified into quintiles using the equal-frequency method: participants were ranked from lowest to highest based on each income measure and divided into five equally sized groups (lowest, lower-middle, middle, upper-middle, and highest), following the quintile classification approach of the National Bureau of Statistics of China [14].

Satisfaction with cancer screening was measured using a 5-point Likert scale, ranging from very satisfied to very dissatisfied. Residents who reported being satisfied or very satisfied with cancer screening services were classified as satisfied, while those who responded neutral, dissatisfied, or very dissatisfied were classified as dissatisfied. This dichotomization was conducted to simplify interpretation.

$$\mathrm{Cancer}\;\mathrm{screening}\;\mathrm{satisfaction}=\frac{{Number}\;{of}\;{people}\;{satisfied}\;{with}\;{cancer}\;{screening}}{{Total}\;{number}\;{of}\;{participants}\;{in}\;{cancer}\;{screening}}\ast100\%$$

To minimize interviewer bias, all interviewers received standardized training and used a structured questionnaire.

### Statistical analysis

The χ² test was applied to analyze the distribution of socioeconomic factors, lifestyle factors, and individual health status among participants who were satisfied or dissatisfied with cancer screening. Both univariate and multivariate logistic regression models were performed to calculate the Odds Ratios (ORs) and corresponding 95% confidence intervals (95% CIs) for each variable. To control for type I error inflation due to multiple comparisons, false discovery rate (FDR) correction was applied. Variables with an FDR < 0.1 in the univariate analysis were included in the multivariate model. Multicollinearity was assessed using variance inflation factors (VIF), with VIF > 10 indicating significant collinearity [15]. The analysis was further stratified by free cancer screening and paid cancer screening. Given that the data were hierarchical, we used multilevel modeling to examine whether provincial-level factors contributed to satisfaction beyond individual-level characteristics as a sensitivity analysis. Three models were constructed for multilevel binary logistic regression analysis. Model 1 was a null model without explanatory variables. Model 2 included individual-level covariates. Model 3 (full) further added provincial-level variables, including GDP per capita, hospital beds per 1,000 population, and number of tertiary hospitals (rescaled per 100-hospital increment for interpretability) in 2022. Provincial-level data were obtained from the National Bureau of Statistics of China (GDP) and the National Health Commission of China (healthcare resources). The intraclass correlation coefficient (ICC) and proportional change in variance (PCV) were estimated to quantify random effects. Model fit was compared using theAkaike information criterion (AIC), Bayesian information criterion (BIC), and likelihood ratio tests. Based on these results, structural equation modeling (SEM) was utilized to conduct a pathway analysis of the various factors associated with residents’ satisfaction with cancer screening. SEM was estimated using the DWLS (diagonally weighted least squares) estimator, which is appropriate for categorical/ordinal indicator variables. Indirect effects were tested via bootstrap resampling with 2,000 iterations. Model fit was considered acceptable if the root mean square error of approximation (RMSEA) was less than 0.08, and the comparative fit index (CFI), goodness-of-fit index (GFI), and normed fit index (NFI) were greater than 0.90 [16].

The analysis was conducted using SPSS version 25.0 and R version 4.5.1. All p-values were derived from two-sided tests with a significance level (α) of 0.05.

## Result

### General information

**Fig. 1:**
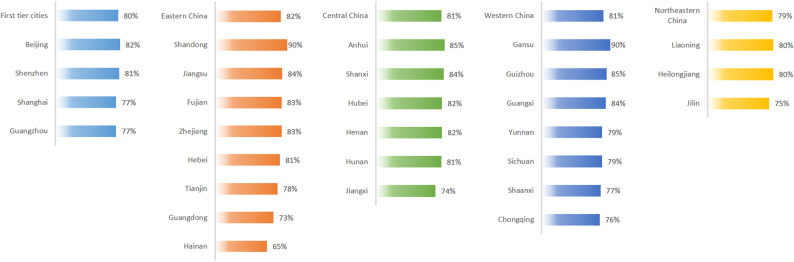
Satisfaction with cancer screening service by regions and provinces of China

The study encompasses 26 provinces in mainland China, covering a total of 85 cities. The overall satisfaction with cancer screening was high at 82.8%. Satisfaction rates varied across provinces, ranging from 67% (Hainan) to 92% (Shandong). Provinces with relatively high satisfaction included Gansu (91%) in western China, Shandong (92%) in eastern China, and Shanxi (87%) in central China. Conversely, provinces with relatively low satisfaction included Hainan (67%) in eastern China, Jiangxi (76%) in central China, and Jilin (75%) in northeastern China. Cancer screening satisfaction among residents across various provinces in China is illustrated in Fig. 1.

The distribution of general demographic characteristics, health behavior patterns, individual health status evaluations, and cancer screening satisfaction is presented in Table 1. Overall, the factors significantly associated with satisfaction in the univariate analysis included gender, smoking, passive smoking, intake of fresh vegetables, intake of fresh fruit, physical activity, family history of cancer, chronic diseases, self-rated health status, and annual household disposable income.

**Table 1:** Basic characteristics of the participants and univariate analysis of their satisfaction with cancer screening (*N* = 5024)

Characteristics	Total (No)	No. of satisfied	No. of dissatisfied	Satisfied (%)	*P* value	FDR-adjusted *P*-value
Gender					0.015	0.033
Male	3260	2729	531	83.7%		
Female	1764	1429	335	81.0%		
Age					0.893	0.95
< 60	3751	3106	645	82.8%		
≥ 60	1273	1052	221	82.6%		
BMI (kg/m^2^)					0.387	0.45
< 18.5	190	160	30	84.2%		
18.5 ≤ BMI < 24	2695	2212	483	82.1%		
≥ 24	2139	1786	353	83.5%		
Education					0.170	0.24
Primary	1191	964	227	80.9%		
Secondary	2682	2238	444	83.4%		
Higher	1151	956	195	83.1%		
Work					0.396	0.45
Unemployed	1560	1286	274	82.4%		
No risk	339	270	69	79.6%		
With risk	3125	2602	523	83.3%		
Marriage					0.181	0.222
Have a spouse	4507	3741	766	83.0%		
Other	517	417	100	80.7%		
Smoking					**<0.001**	**0.002**
No	3819	3199	620	83.8%		
Yes	1205	959	246	79.6%		
Passive smoking					**0.003**	**0.008**
No	2699	2273	426	84.2%		
Yes	2325	1885	440	81.1%		
Alcohol consumption					0.946	0.95
No	4487	3713	774	82.8%		
Yes	537	445	92	82.9%		
Fresh vegetable intake					**<0.001**	**0.002**
<2.5 kg per week	1436	1147	289	79.9%		
≥2.5 kg per week	3588	3011	577	83.9%		
Fresh fruit intake					**<0.001**	**<0.001**
<1.25 kg per week	2023	1611	412	79.6%		
≥1.25 kg per week	3001	2547	454	84.9%		
Physical activity					**<0.001**	**<0.001**
No	1903	1515	388	79.6%		
Yes	3121	2643	478	84.7%		
Family history of cancer					**0.016**	**0.03**
No	4162	3469	693	83.3%		
Yes	862	689	173	79.9%		
Chronic diseases status					**<0.001**	**<0.001**
No	3582	3028	554	84.5%		
Yes	1442	1130	312	78.4%		
Self-rated health status					**<0.001**	**<0.001**
Very poor	51	35	16	68.6%		
Poor	231	161	70	69.7%		
Fair	1355	1006	349	74.2%		
Good	2200	1876	324	85.3%		
Excellent	1187	1080	107	91.0%		
Personal annual disposable income (10,000 CNY)					0.137	0.21
Lowest income group	1320	1066	254	80.8%		
Lower-middle income group	899	764	135	85.0%		
Middle income group	811	670	141	82.6%		
Upper-middle income group	1146	939	207	81.9%		
Highest income group	848	719	129	84.8%		
Annual household disposable income (10,000 CNY)					**0.024**	**0.041**
Lowest income group	1077	870	207	80.8%		
Lower-middle income group	1067	880	187	82.5%		
Middle income group	944	782	162	82.8%		
Upper-middle income group	944	788	156	83.5%		
Highest income group	992	838	154	84.5%		

Significant *P* values appear in bold

These factors were utilized as independent variables in a multivariate logistic regression analysis. All VIF values were below 3, indicating no severe multicollinearity among the independent variables. Physical activity remained significantly associated with participants’ overall satisfaction with cancer screening, with physically active participants showing higher satisfaction (OR = 1.27, 95% CI: 1.08–1.48). Similarly, self-rated health status remained significantly associated, with better self-rated health linked to higher satisfaction (OR for the excellent health status = 4.51, 95% CI: 2.35–8.65, *P* for trend < 0.001). However, the positive association between fresh vegetable intake and screening satisfaction (OR = 1.19, 95% CI: 1.01–1.42) was no longer significant after FDR correction.

When cancer screening was stratified into free and paid screening, physical activity remained significantly associated with satisfaction with free screening (OR = 1.45, 95% CI: 1.14–1.84). However, participants with a family history of cancer reported lower satisfaction with free screening (OR = 0.63, 95% CI: 0.48–0.84). For paid screening, better self-rated health (OR for excellent health status = 3.20, 95% CI: 1.62–6.34; trend *P* < 0.001) continued to be associated with higher satisfaction. Although physical activity was associated with satisfaction with paid screening (OR = 1.22, 95% CI: 1.02–1.44), this association was no longer significant after FDR correction (see Table 2).

**Table 2:** Logistic regression for the factors associated with participants’ satisfaction with cancer screening

Risk factors	Overall		Free screening		Paid screening	
	OR (95%CI)	*P* value	OR (95%CI)	*P* value	OR (95%CI)	*P* value
Gender						
Female	1.00 (REF)		1.00 (REF)		1.00 (REF)	
Male	0.92 (0.74–1.15)	0.488	1.05 (0.71–1.56)	0.806	1.07 (0.84–1.37)	0.591
Smoking						
No	1.00 (REF)		1.00 (REF)		1.00 (REF)	
Yes	0.86 (0.67–1.09)	0.212	0.97 (0.62–1.52)	0.883	0.87 (0.66–1.14)	0.323
Passive smoking						
No	1.00 (REF)		1.00 (REF)		1.00 (REF)	
Yes	0.87 (0.74–1.01)	0.074	0.87(0.68–1.11)	0.267	0.86 (0.73–1.03)	0.096
Fresh vegetable intake						
< 2.5 kg per week	1.00 (REF)		1.00 (REF)		1.00 (REF)	
≥ 2.5 kg per week	1.19 (1.01–1.42)	0.049*	1.08 (0.82–1.43)	0.563	1.13 (0.94–1.38)	0.203
Fresh fruit intake						
< 1.25 kg per week	1.00 (REF)		1.00 (REF)		1.00 (REF)	
≥ 1.25 kg per week	1.11 (0.94–1.32)	0.210	1.10 (0.85–1.44)	0.466	1.15 (0.96–1.39)	0.133
Physical activity						
No	1.00 (REF)		1.00 (REF)		1.00 (REF)	
Yes	1.27 (1.08–1.48)	0.003**	1.45 (1.14–1.84)	0.003**	1.22 (1.02–1.44)	0.026*
Family history of cancer						
No	1.00 (REF)		1.00 (REF)		1.00 (REF)	
Yes	0.87 (0.72–1.05)	0.145	0.63 (0.48–0.84)	0.002**	1.04 (0.84–1.28)	0.726
Chronic diseases status						
No	1.00 (REF)		1.00 (REF)		1.00 (REF)	
Yes	1.080 (0.90–1.30)	0.405	1.07 (0.81–1.42)	0.637	1.11 (0.91–1.36)	0.314
Self-rated health status						
Very poor	1.00 (REF)		1.00 (REF)		1.00 (REF)	
Poor	1.06 (0.55–2.05)	0.859	0.99 (0.26–3.88)	0.995	0.92 (0.47–1.83)	0.818
Fair	1.31 (0.71–2.42)	0.390	0.84 (0.24–3.02)	0.791	1.02 (0.54–1.92)	0.963
Good	2.63 (1.40–4.92)	0.002**	1.62 (0.45–5.88)	0.464	1.87 (0.97–3.58)	0.060
Excellent	4.51 (2.35–8.65)	<0.001**	3.42 (0.91–12.84)	0.069	3.20 (1.62–6.34)	0.001**
*Trend test p-value*		<0.001**		<0.001**		<0.001**
Annual household disposable income (10,000 CNY)						
Lowest income group	1.00 (REF)		1.00 (REF)		1.00 (REF)	
Lower-middle income group	1.01 (0.81–1.27)	0.902	0.93 (0.64–1.35)	0.708	1.10 (0.86–1.42)	0.431
Middle income group	0.98 (0.77–1.23)	0.846	0.97 (0.66–1.42)	0.877	1.01 (0.78–1.31)	0.932
Upper-middle income group	1.02 (0.80–1.30)	0.861	0.89 (0.61–1.31)	0.560	1.09 (0.84–1.42)	0.527
Highest income group	0.99 (0.77–1.26)	0.930	0.78 (0.53–1.14)	0.197	1.09 (0.83–1.44)	0.526
* Trend test p-value*		0.997		0.729		0.909

* *P* < 0.05; ** FDR-adjusted *P* < 0.05. Significant *P* values appear in bold

### Provincial-level contextual factors and regional disparities

Given that individuals were nested within provinces, we performed a two-level random-intercept logistic regression. The null model showed that province-level differences accounted for 1.4% of the total variance (ICC = 0.014). Adding individual-level covariates (Model 2) significantly improved model fit (LR χ²(13) = 200.77, *P* < 0.001) and explained 20.7% of the provincial variance (PCV = 20.7%). However, further adding GDP per capita, hospital beds per 1,000 population, and tertiary hospitals did not significantly improve fit (LR χ²(3) = 2.73, *P* = 0.435). Model 2 had the lowest AIC (4437) and was confirmed as the final model (see Table 3).

**Table 3:** Multilevel logistic regression results for overall satisfaction: Provincial-level effects and model fit

	Model 1 (null model)	Model 2 (individual-level factors)	Model 3 (Provincial-level factors)
Provincial-level variables			
GDP per capita (10,000 CNY)	—	—	1.002 (0.976–1.029)
Hospital beds (per 1,000)	—	—	1.031 (0.897–1.185)
Tertiary hospitals (per 100 increase)			1.012 (0.996–1.029)
Random effects			
variance (SD)	0.046 (0.215)	0.037 (0.192)	0.029 (0.170)
ICC	1.4%	1.1%	0.9%
PCV	Ref.	20.7%	37.8%
Model fitness			
AIC	4612	4437	4440
BIC	4625	4535	4558
Log likelihood	−2304	−2204	−2202
LR test (vs. previous)	—	χ²(13) = 200.77***	χ²(3) = 2.73

OR (95% CI) shown for provincial-level variables

*ICC*  intraclass correlation coefficient, *PCV*  proportional change in variance, *LR*  likelihood ratio test

***p < 0.001

### The chain-mediating effects of self-rated health and chronic disease status on the relationship between economic status and cancer screening satisfaction

In this study, a hypothesized model was developed in which annual household disposable income served as an indicator of economic status, while self-rated health, chronic disease status, and screening service expectations (intended institutional level) functioned as mediators, collectively associated with cancer screening satisfaction. The analyses were stratified into two groups: free cancer screening and paid cancer screening (supplementary Tables 1 and 2). Model fit indices for both groups met standard acceptable thresholds (supplementary Table 3).

**Fig. 2:**
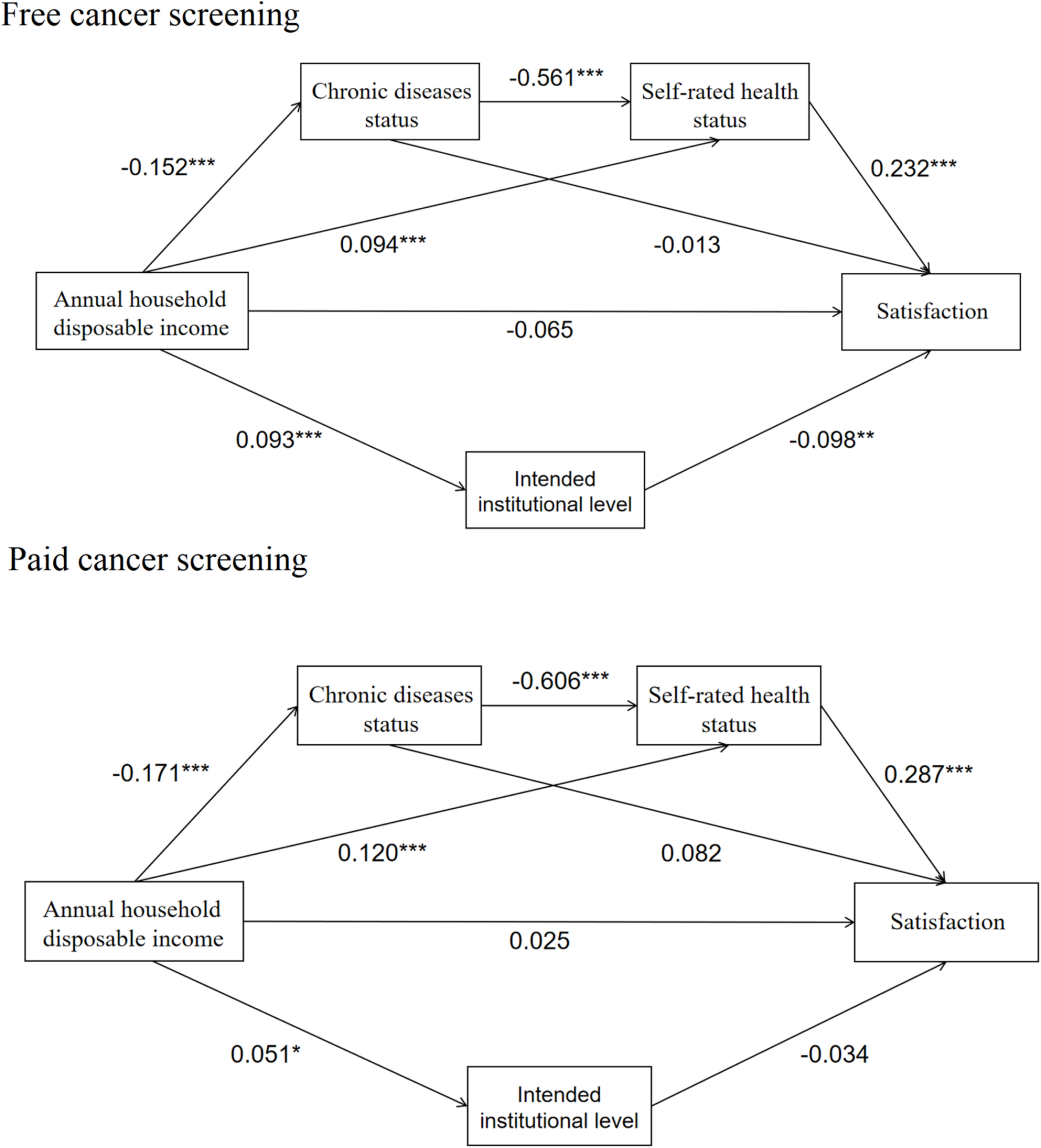
Pathways of factors associated with satisfaction with free and paid cancer screening

As shown in Fig. 2, chronic disease status and self-rated health status exhibit a chain mediation effect on the relationship between annual household disposable income and satisfaction with free and paid cancer screenings, with a 65.4% total percentage of mediation for paid screening. In addition, screening service expectations (intended institutional level) negatively mediated the relationship between annual household disposable income and satisfaction with free cancer screening.

## Discussion

In this study, overall satisfaction with cancer screening was high at 82.8%, suggesting that current screening services meet the expectations of participants—a finding consistent with those of other studies [8]. Higher satisfaction with cancer screening may be attributed to physicians’ interpersonal communication skills and timeliness of services [17]. However, there are still regional differences in satisfaction across China, with even larger differences within the same region. These differences might result from the unequal quality of health service in China [18].

Satisfaction with the cancer screening is related to participants’ lifestyles in our study. Participants with higher intake of fresh vegetables and fruit reported greater satisfaction with cancer screening. This association may be explained by health behavior clustering—individuals with healthier dietary habits often possess greater health consciousness and engagement in preventive care [19], which may translate into more positive attitudes toward preventive services and higher satisfaction with healthcare encounters. In addition, physically active individuals exhibit higher satisfaction with cancer screening. This finding aligns with previous research indicating that health-promoting lifestyles and health promotion interventions in healthcare settings are positively associated with patient satisfaction [20]. One possible explanation is that individuals who had a healthy lifestyle may be more attentive to their health and perceive the time and financial costs associated with cancer screening programs as worthwhile [21]. Consequently, their satisfaction tends to be higher.

People with a family history of cancer have lower satisfaction with free screening programs, which is consistent with previous findings [22]. First-degree relatives of cancer patients tend to have greater awareness of screening, a stronger willingness to undergo screening, and consequently a higher demand for comprehensive screening services. As a result, they might be less satisfied with the basic and free screening measures. The hereditary nature of cancer, combined with its high incidence and mortality rates, imposes a significant psychological burden on people with a family history of cancer [23], which likely contributes to their decreased satisfaction with screening programs.

In addition to these individual-level determinants, we used multilevel modeling to examine whether provincial-level contextual factors contributed to regional satisfaction disparities. The null model showed that province-level differences accounted for only 1.4% of the total variance in satisfaction, while adding individual-level covariates explained 20.7% of the provincial variance, suggesting individual characteristics as major contributing factors. Additionally, none of the measured provincial-level variables (GDP per capita, hospital bed density, or number of tertiary hospitals) were significantly associated with satisfaction. This may reflect narrowed inter-provincial disparities in healthcare infrastructure [24] or insufficient granularity of provincial-level indicators, as aggregate measures may mask within-province heterogeneity [25].

This study found that individuals with chronic diseases exhibited lower satisfaction with cancer screening, consistent with previous research showing that patients with multiple chronic conditions have significantly lower odds of reporting satisfactory healthcare experiences [26]. However, after adjusting for covariates in multivariate analysis, the effect was attenuated to a null association. This apparent inconsistency is resolved through structural equation modeling. In the paid screening group, the direct path from chronic disease to satisfaction was non-significant, while the effect operated primarily through an indirect pathway: higher annual household disposable income was associated with lower chronic disease burden, which in turn was linked to better self-rated health, and ultimately to higher cancer screening satisfaction. This income→chronic disease→self-rated health→satisfaction pathway suggests that the lower satisfaction observed among chronic disease patients in univariate analysis is largely explained by their lower income and poorer self-rated health.

Additionally, our study found that better self-rated health status was associated with higher satisfaction with cancer screening. Self-rated health is a comprehensive indicator integrating objective health status, functional capacity, and psychological well-being, and it has consistently been shown to be a strong predictor of healthcare satisfaction [27]. Individuals with better self-rated health tend to perceive their healthcare experiences more positively, resulting in higher satisfaction with preventive services such as cancer screening [28]. Self-rated health partially mediated the relationship between annual household disposable income and screening satisfaction in both free and paid settings [29, 30]. Disparities in household income affect living standards, consumption expenditure, and healthcare expenditure, thereby associated with health status [31]. Consequently, good household finances contribute not only to improving family health but also to increasing resident satisfaction with cancer screening through enhanced self-rated health.

Our findings also suggest that screening service expectations mediate the association between annual household disposable income and satisfaction with cancer screening in free screening programs. Residents preferred to receive screening services at higher-level provincial hospitals and sought a higher level of service. The tier of the screening facility is an important factor affecting expectations for cancer screening services, with residents tending to prefer higher quality of medical care in economically developed cities and at high-level hospitals [32]. However, this preference for higher-level institutions may paradoxically lead to lower satisfaction, as the gap between elevated expectations for quality care and the actual service experience in busy, high-tier facilities often leads to reduced patient satisfaction [33].

The findings of this study have important public health implications. To improve satisfaction with cancer screening, interventions should integrate chronic disease management for low-income populations, provide health education and psychological support for those with poor self-rated health, and implement tailored strategies for free versus paid screening programs. These strategies include enhancing accessibility and delivering family history-specific risk communication for free screening, as well as improving service quality and expectation management for screening.

### Strength and limitations

Our study has several notable strengths. The use of nationwide survey data enhances the representativeness of the results and supports generalizability of our findings. Additionally, the structural equation model analysis further explored the potential mechanism for satisfaction. Nonetheless, our study has several limitations. First, as a cross-sectional study, it cannot establish causal relationship for the factors examined. Second, recall bias may persist due to self-reported data. Third, the satisfaction measured in this study specifically pertains to participants’ general satisfaction with cancer screening services and does not involve the quality or technical performance of the specific hospitals.

## Conclusions

In summary, the overall satisfaction with cancer screening among participants in China is high, particularly in Shandong and Gansu provinces. Satisfaction with cancer screening was primarily driven by individual-level factors, with minimal provincial-level differences observed. Participants’ physical activity, chronic disease and self-rated health status all affect their satisfaction with cancer screening. Household income indirectly influences cancer screening satisfaction through its association with reduced burden of chronic disease and improved self-rated health. These findings highlight the need for targeted interventions that prioritize low-income populations, integrate chronic disease management, and address negative health perceptions, while also adopting differentiated strategies for free versus paid screening programs.

## Supplementary Information

Supplementary Material 1.

Supplementary Material 2.

## Data Availability

The raw data supporting the conclusions of this article will be made available by the corresponding authors, upon reasonable request.
