# Supplementary material for: Satisfaction with cancer screening programs and its associated factors: a cross-sectional study in 26 provinces across China
Source: BMC Public Health. 2026 May 21;26:2143. doi: 10.1186/s12889-026-27706-5 (PMC13366819; doi:10.1186/s12889-026-27706-5)
Supplement: Supplementary file 2 — Supplementary Material 2. [file 12889_2026_27706_MOESM2_ESM.docx]

**Supplementary Table 1. Pathway analysis for the factors associated with satisfaction with free cancer screening in China**

| Pathways | B | β | S.E. | *P* | Mediation proportion (%) |
| --- | --- | --- | --- | --- | --- |
| Annual household disposable income → chronic disease status→self-rated health→satisfaction | 0.014 | 0.020 | 0.004 | **<0.001** | **-66.7%** |
| Annual household disposable income → self-rated health → satisfaction | 0.015 | 0.022 | 0.005 | **0.002** | **-71.4%** |
| Annual household disposable income →chronic disease status → satisfaction | 0.001 | 0.002 | 0.006 | 0.837 | -4.8% |
| Annual household disposable income →intended institutional level → satisfaction | -0.006 | -0.009 | 0.003 | **0.030** | **28.6%** |
| Total Indirect Effect | 0.024 | 0.034 | 0.006 | **<0.001** | **-114.3%** |
| Direct Effect | -0.045 | -0.065 | 0.024 | 0.069 | 214.3% |
| Total Effect | -0.021 | -0.031 | 0.024 | 0.366 | 100% |

Note: B: unstandardized coefficient; β: standardized coefficient

**Supplementary Table 2. Pathway analysis for the factors associated with satisfaction with paid cancer screening in China**

| Pathways | B | β | S.E. | *P* | Mediation proportion (%) |
| --- | --- | --- | --- | --- | --- |
| Annual household disposable income → chronic disease status→self-rated health→satisfaction | 0.021 | 0.030 | 0.004 | **<0.001** | 40.4% |
| Annual household disposable income → self-rated health → satisfaction | 0.024 | 0.034 | 0.005 | **<0.001** | 46.2% |
| Annual household disposable income →chronic disease status → satisfaction | -0.010 | -0.014 | 0.006 | 0.078 | -19.2% |
| Annual household disposable income →intended institutional level → satisfaction | -0.001 | -0.002 | 0.001 | 0.283 | -1.9% |
| Total Indirect Effect | 0.034 | 0.049 | 0.006 | **<0.001** | 65.4% |
| Direct Effect | 0.018 | 0.025 | 0.019 | 0.321 | 34.6% |
| Total Effect | 0.052 | 0.074 | 0.017 | **0.003** | 100% |

Note: B: unstandardized coefficient; β: standardized coefficient
